# Microbiota Succession of Whole and Filleted European Sea Bass (*Dicentrarchus labrax*) during Storage under Aerobic and MAP Conditions via 16S rRNA Gene High-Throughput Sequencing Approach

**DOI:** 10.3390/microorganisms10091870

**Published:** 2022-09-19

**Authors:** Faidra Syropoulou, Dimitrios A. Anagnostopoulos, Foteini F. Parlapani, Evangelia Karamani, Anastasios Stamatiou, Kostas Tzokas, George-John E. Nychas, Ioannis S. Boziaris

**Affiliations:** 1 Laboratory of Marketing and Technology of Aquatic Products and Foods, Department of Ichthyology and Aquatic Environment, School of Agricultural Sciences, University of Thessaly, Fytokou Street, 38446 Volos, Greece; 2 Laboratory of Microbiology and Biotechnology of Foods, Department of Food Science and Human Nutrition, Agricultural University of Athens, Iera Odos 75, 11855 Athens, Greece; 3 AVRAMAR Aquaculture SA, 19,3 km Markopoulou–Paiania Ave. 19002 Paiania, Greece

**Keywords:** fish, sea bass, storage, spoilage, microbiota, bacterial communities, Specific Spoilage Organisms, High-Throughput Sequencing, 16S rRNA gene

## Abstract

In the present work, the profiles of bacterial communities of whole and filleted European sea bass (*Dicentrarchus labrax*), during several storage temperatures (0, 4, 8 and 12 °C) under aerobic and Modified Atmosphere Packaging (MAP) conditions, were examined via the 16S rRNA High-Throughput Sequencing (HTS) approach. Sensorial attributes were also assessed to determine products’ shelf-life. Results indicated that shelf-life was strongly dependent on handling, as well as on temperature and atmosphere conditions. HTS revealed the undisputed dominance of *Pseudomonas* from the very beginning and throughout storage period in the majority of treatments. However, a slightly different microbiota profile was recorded in MAP-stored fillets at the middle stages of storage, which mainly referred to the sporadic appearance of some bacteria (e.g., *Carnobacterium*, *Shewanella*, etc.) that followed the dominance of *Pseudomonas*. It is noticeable that a major difference was observed at the end of shelf-life of MAP-stored fillets at 12 °C, where the dominant microbiota was constituted by the genus *Serratia*, while the relative abundance of *Pseudomonas* and *Brochothrix* was more limited. Furthermore, at the same temperature under aerobic storage of both whole and filleted fish, *Pseudomonas* almost co-existed with *Acinetobacter*, while the presence of both *Erwinia* and *Serratia* in whole fish was noteworthy. Overall, the present study provides useful information regarding the storage fate and spoilage status of whole and filleted European sea bass, suggesting that different handling and storage conditions influence the shelf-life of sea bass by favoring or delaying the dominance of Specific Spoilage Organisms (SSOs), affecting in parallel to some extent the formation of their consortium that is responsible for products’ sensorial deterioration. Such findings enrich the current knowledge and should be used as a benchmark to develop specific strategies aiming to delay spoilage and thus increase the products’ added value.

## 1. Introduction

The dietary trends that have been formed in the last decades have strongly upgraded the role of seafood globally, making the aquatic products a vital part of several diets, worldwide [1]. The increasing demand for seafood has forced the aquaculture industry to rapidly increase production [2] in order to meet market needs and thus ensure its viability and growth. There are several fish species produced worldwide, each of which possesses a particular socio-economic significance for the producing country, it being closely linked to the economy, regional tradition and local diet. In Greece, fish production was a major counterweight in the economic crisis era, since the Hellenic aquaculture industry is always one of the top producers in European Union, as well as in the wider Mediterranean basin, reaching a production of more than 100 K tonnes annually [3].

European sea bass (*Dicentrarchus labrax*) belongs to an important group of seafood with an extremely commercial reach, being one of the most popular fish species in great demand, worldwide [4]. It is noticeable that from the very beginning of the production’s officially recorded existence, Greece has been among the top three producers of sea bass [5], making this fish and its related products a significant pillar in the country’s primary production and exports. Based on the aforementioned, it is reasonable that sea bass’ significant importance stimulates the scientific interest for a thorough research in a wide range of fields. Among others, the study of its spoilage status and the determination of its shelf-life during storage conditions are of great importance to develop targeted/specific strategies that will delay spoilage, extend shelf-life and, consequently, increase the added value of the product [6].

As all seafood products, seabass is among the most perishable foodstuffs, worldwide. It is well-known that fresh fish spoils rapidly during storage, due to the prevalence of a specific bacterial consortium, the so-called Specific Spoilage Organisms (SSOs), which are able to deteriorate the product’s sensorial attributes via their metabolic activity, leading to the rejection of the product [7,8]. The latter constitute a major issue with great economic loss for the industry, since it is estimated that a large number of fish produced (~30%) is discarded before reaching the final recipient, which is the consumer [6]. Τhe fact that this high amount of products does not fit into the normal commerce cycle, the so-called “from farm to fork”, in combination with the fact that seafood deteriorates rapidly under storage conditions, led the scientific community to deeply approach the spoilage phenomenon, studying the microorganisms and the mechanisms that are responsible for it.

Even though our knowledge about seafood spoilage has been greatly enriched in the last two decades, further research is totally imperative, since spoilage constitutes a complex phenomenon that is influenced by several factors at the pre- and post-harvesting stages (handling, processing, storage conditions, etc.) [8,9,10,11]. For instance, the SSOs composition is influenced by different handling (e.g., whole, gutted and filleted fish), as well as storage conditions (air, MAP, etc.), being in parallel closely related to the pre-storage microbiota (initial microbiota) and the microbial interaction during storage [6]. Beyond SSOs, a smaller part of SSOs, the so-called ephemeral spoilage organisms (ESOs), may be favored under specific storage conditions (e.g., high temperatures under MAP conditions), prevail over the rest of the microbiota, and produce several metabolites responsible for off-flavors/odors, resulting in parallel in the rapid organoleptic rejection [12,13,14].

To tackle such problems, food microbiologists attempt to develop novel strategies aiming to retard spoilage phenomenon and thus extend seafood shelf-life. For this, a holistic approach, regarding the microbiota present, how this microbiota is changing under several storage conditions and, consequently, how storage conditions affect the SSOs consortium formation, is a pre-requisite. The increase in knowledge about the aforementioned will help tackle the spoilage phenomenon rationally and thus maximize the added value and the quality of seafood. Nowadays, the advent of High-Throughput Sequencing (HTS) techniques and, more specifically, the 16S rRNA metabarcoding analysis made the study of microbial communities an easy task, providing useful information about the microorganisms that may be responsible for food/seafood spoilage [6,11,15,16]. This technique has revolutionized the field of microbiological research [17], being a benchmark in modern studies, as well as an important tool for an in-depth approach in the field of food microbiology. The aim of the present study was to determine the microbiota profile of whole and filleted European sea bass (*Dicentrarchus labrax*) stored in various conditions throughout shelf-life by HTS analysis.

## 2. Materials and Methods

### 2.1. Provision, Storage and Sampling of Samples

Whole and filleted European seabass (*Dicentrarchus labrax*) of approximately 500 g and 200 g, respectively, were provided by Avramar Aquaculture SA (Greece) in November 2019. Fish were harvested (Day 0) from various farms in Greece and transferred to the Avramar Aquaculture SA processing plant the same day wherein handled as whole fish or filleted. Both products were packed separately in insulated boxes with melted ice. The MAP products were packed in trays with BDF 8050F low oxygen transmission rate film (Crovac-Sealed Air Ltd., Athens, Greece). Then, the samples were transferred to the Laboratory of Marketing and Technology of Aquatic Products and Foods (University of Thessaly, Volos), where they were stored at different conditions as described in Table 1. It should be mentioned that regarding storage at high temperature such as 8 or even 12 °C, it is of great importance to examine the microbiota profile of seafood stored under abuse temperature conditions. The refrigerators of markets and/or restaurants are constantly opening and closing, resulting in temperature rising to high levels. Thus, in such kind of experiments, the worst-case scenario should also be considered.

### 2.2. Evaluation of Samples Sensory Rejection

Sensory evaluation aimed to determine samples’ rejection time point. The analysis was applied as described by Syropoulou et al. [18] and took place every day (every 20–24 h) for samples stored at 0, 4 and 8 °C, while at 12 °C sampling was applied every 10–12 h. More specifically, five trained panelists evaluated the sensory attributes (e.g., skin appearance, firmness and odor of flesh) of the whole fish, scoring each parameter on a descriptive hedonic scale (5, 4, 3 and 2 corresponding to the categories E, A, B and C, respectively; 5 being the highest quality score and 1 the lowest) according to [19]. Average score below 3 was attributed to sample’s rejection. Additionally, the same scale was used for fillets assessment, scoring several parameters in appearance (translucent, glossy, natural color, opaque, dull, discolored) and odor (marine, fresh, neutral, sour, stale, spoiled, putrid), and firmness, and an average score of 3 was taken as the score for minimum acceptability, while an average score below 3 was attributed to sample’s rejection.

### 2.3. 16S Metabarcoding Analysis

#### 2.3.1. Samples Preparation and DNA Extraction

Samples preparation for DNA extraction was applied following the procedure described by Syropoulou et al. [18]. Specifically, a total of 25 g of pooled samples from four fish or fish fillets was transferred to stomacher bags with 225 mL sterile saline solution (0.85% *w*/*v*) and homogenized for 4 min in a Stomacher. All the volume of homogenized suspension was transferred to sterile tubes and minor centrifuged at 136× *g* for 5 min at 20 °C (NF 400R bench top refrigerated centrifuge, Nuve, Turkey) to remove any particles. Then, the supernatant was carefully transferred to new sterile tubes and centrifuged at 2067× *g* for 15 min at 20 °C. The resulting pellet was resuspended in 1 mL of sterile deionized H_2_O, from which 200 μL were used. Bacterial DNA extraction was applied by the NucleoSpin Tissue kit (Macherey-Nagel GmbH & Co. KG, Düren, Germany), following manufacturer’s instructions. Finally, DNA concentration was determined on a nanodrop Quawell UV–VIS Spectrophotometer Q5000 (Quawell Technology, Inc., San Jose, CA, USA).

#### 2.3.2. Library Preparation, High-Throughput Sequencing and Bioinformatic Analysis

High-Throughput Sequencing was applied using the primers 27F (AGRGTTTGATCMTGGCTCAG) and 519Rmodbio (GWATTACCGCGGCKGCTG) to amplify the V1-V3 region of 16S rRNA gene. Samples incurred in a 30 cycles Polymerase Chain Reaction (PCR) using the HotStarTaq Plus Master Mix Kit (Qiagen, Valencia, CA, USA). PCR conditions were (a) 95 °C for 5 min, followed by (b) 30 cycles of 95 °C for 30 s, 53 °C for 40 s and 72 °C for 1 min and (c) a final elongation at 72 °C for 10 min. Finally, the resulting amplicons were pooled in equal concentrations and sequenced on a MiSeq Illumina platform (San Diego, CA, USA) following the manufacturer’s protocol.

Bioinformatic analysis was applied using the MR DNA ribosomal and functional gene analysis pipeline (www.mrdnalab.com; accessed on 20 July 2022), MR DNA, Shallowater, TX, USA). High-quality sequences (≥Q25) were retained and underwent quality filtering, dereplication and denoising as described previously [18,20]. Unique sequences identified with sequencing or PCR point errors (low quality reads and/or read errors) were removed, followed by chimera checking. The resulting zOTUs (zero-radius Operational Taxonomic Units) were taxonomically classified using BLASTn against a curated database deriving from the National Center for Biotechnology Information (NCBI) (www.ncbi.nlm.nih.gov; accessed on 20 July 2022) and generated into percentage (%) of relative abundances at different taxonomic levels (from phylum to genus). Alpha and beta diversities were estimated according to previous works [21,22,23,24] using the Quantitative Insights Into Microbial Ecology 2 (Qiime 2) pipeline [25]. A rarefaction to 5000 sequences was implemented using the DADA2 algorithm and plotted with 10 sampling depths. For beta diversity, a Principal Coordinate Analysis (PCoA) was applied using the weighted UniFrac distance. Finally, raw sequences were deposited in the National Centre for Biotechnology Information (NCBI), under the Bioproject PRJNA861973.

### 2.4. Statistical Analysis

Differences of mean values in sensory evaluation were statistically tested. The data were subjected to Analysis of Variance (ANOVA) followed by Tukey post hoc test using the IBM^®^ SPSS^®^ statistics 19 software (SPSS Inc., Chicago, IL, USA) and a probability level of *p* ≤ 0.05 was considered statistically significant.

## 3. Results

### 3.1. Sensory Evaluation of Whole and Filleted Sea Bass

At the initial stage of storage (0 h), the general appearance of both whole fish and fillets was excellent (Grade 5), exhibiting shiny skin, firm flesh, marine odors and convex eyes with black pupils as well as marine odor of gills. However, the sensory characteristics deteriorated rapidly with the passage of storage time. At the rejection point (Grade below 3) the skin had slightly lost its brightness, and the eyes were flat with translucent pupils, while the unpleasant odor produced was the most obvious feature that testified the spoilage. Each treatment reached the minimum acceptability level at different time points. More specifically, according to Table 2, the end of shelf-life was defined at 281, 185, 65 and 41 h for whole fish stored under aerobic conditions at 0, 4, 8 and 12 °C, respectively. However, an earlier end of shelf-life was recorded in air-stored fish fillets. The end of shelf-life for those samples was determined at 143, 70, 58 and 34 h at storage temperatures of 0, 4, 8 and 12 °C, respectively. On the other hand, as expected, the MAP-stored fillets recorded an extended shelf-life, since the minimum acceptability level at 0, 4, 8 and 12 °C, was assessed at 585, 305, 185 and 113 h, respectively.

### 3.2. Microbial Diversity of Whole and Filleted Seabass

According to the bioinformatic analysis, a total of 741,010 raw reads were obtained from all samples, and after the necessary quality control (filtering, denoising and chimera checking), 375,449 of them were retained (average of about 12,515 per sample) (Table 3). Those reads corresponded to a total of 1764 observed features (which ranged from 24 to 135). It is crucial to point out that the characterization of bacterial diversity is considered very reliable, since the depth of rarefaction applied (5000) was found to be sufficiently satisfactory (e.g., Shannon–Wiener Index curves had already reached a plateau at ~500 sequences in all samples) (Figure S1).

**Figure 1:**
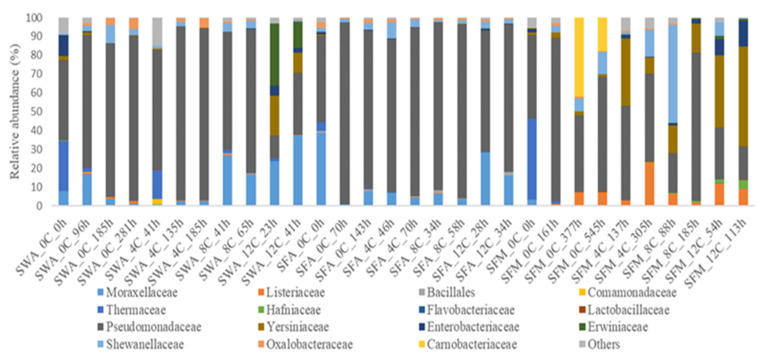
Relative abundance (%) of bacterial families of whole (SW) and filleted sea bass (SF) at intervals of storage hours (h), at various temperatures (C) under air (A) and MAP (M) conditions, as revealed by 16S rRNA metabarcoding analysis.

The results of 16S rRNA metabarcoding analysis regarding the bacterial profiles of different treatments at phylum and family levels are given in Figure S2 and Figure 1, respectively. In general, it should be noted that the composition of microbial communities was quite similar among the majority of treatments, even though in some cases, storage conditions affected to some extent the microbiota formation at advanced period.

More specifically, the dominance of bacteria belonging to Proteobacteria phylum was indisputable in all cases throughout storage time, reaching mean levels of about 65% and 93% for whole and filleted fish, respectively, at the beginning of storage (0 h). At the middle and the end of shelf-life, the relative abundance of Proteobacteria exhibited levels of more than 97% in the majority of the treatments, with some exceptions. For instance, in advanced storage and the end of shelf-life (377 h and 545 h, respectively) of MAP-stored fillets at 0 °C, the high presence of Firmicutes was found to be close to Proteobacteria, reaching relative abundance of about 49% and 51%, respectively. Similar findings, even though to a lesser extent, were observed for the MAP-stored fillets under all the studied temperatures, since the presence of Firmicutes was always remarkable, being the second most abundant bacterial phylum. Other phyla that were detected to a lesser extent and always at the initial stages of storage were Actinobacteria and Deinococcus-Thermus, while Bacteroidetes were detected in traces (<1%), in all cases.

At family level, Pseudomonadaceae was the dominant player in all samples stored under aerobic conditions in both whole and filleted fish. However, it is worth noting that this family had already recorded the highest relative abundance from the initial stage of storage (0 h). Exception to this dominance was observed in whole fish samples stored aerobically at 12 °C, where at the end of shelf-life (41 h), the family Pseudomonadaceae co-existed in perfect balance with that of Moraxellaceae (relative abundance of about 33% and 37%, respectively), while the presence of both Erwiniaceae (~14% relative abundance) and Yersiniaceae (~11% relative abundance) is remarkable. The latter was the predominant bacterial family at the end of shelf-life of fillets stored at 12 °C under MAP conditions (~53% relative abundance), while the presence of Pseudomonadaceae was more limited (~18% relative abundance), followed by Enterobacteriaceae (~14% relative abundance), Listeriaceae (~9% relative abundance) and Hafniaceae (~5% relative abundance). Finally, the profile of MAP-stored fillets at 0 °C should be emphasized because at the middle and the end of shelf-life (377 h and 545 h, respectively), the bacterial composition was consisted mainly by the family Pseudomonadaceae, while the presence of Carnobacteriaceae was also noteworthy (relative abundance of about 42% and 18% at 377 h and 545 h, respectively).

**Figure 2:**
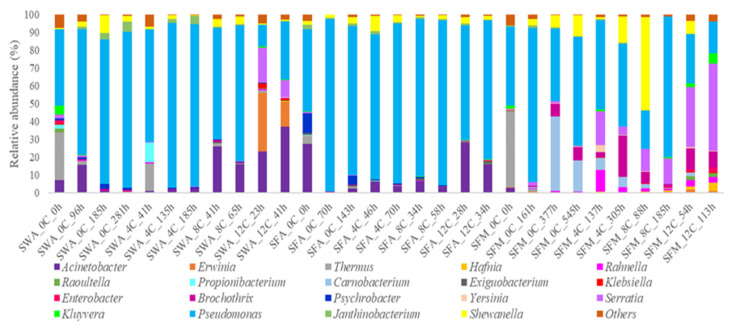
Relative abundance (%) of bacterial genera of whole (SW) and filleted sea bass (SF) at intervals of storage hours (h), at various temperatures (C) under air (A) and MAP (M) conditions, as revealed by 16S rRNA metabarcoding analysis.

At genus level (Figure 2), the initial microbiota (0 h) consisted mainly by the genera *Pseudomonas* and *Thermus* in both whole and filleted samples. In the latter, the presence of both *Acinetobacter* and *Psychrobacter* was also at noteworthy levels (28% and 11% relative abundance, respectively). At advanced storage period and the end of shelf-life of each product, the genus *Pseudomonas* was by far the most dominant bacterial genus under aerobic conditions in both whole and filleted fish. However, the microbiota of whole fish stored aerobically at 12 °C was constituted by a slightly different profile, since *Pseudomonas* co-dominated with *Acinetobacter* (33% and 38% relative abundance, respectively), followed by the strong presence of *Erwinia* (~14% relative abundance), while *Serratia* was also found at a lower but remarkable relative abundance (~10%). It is also crucial to mention that the latter genus predominated at the end of shelf-life (113 h) of fish fillets stored at 12 °C under MAP conditions, recording a relative abundance of about 49%. On the other hand, at the end of shelf-life (545 h) of MAP-stored fillets at 0 °C, the dominant microbial composition constituted by the genus *Pseudomonas* (~61% relative abundance), followed by *Carnobacterium* (~18% relative abundance), the presence of which was recorded only under these conditions. As for MAP-stored fillets at 4 °C and 8 °C, in the former samples, the dominance of *Pseudomonas* (~47% relative abundance) was followed by *Brochothrix* and *Shewanella* (~23% and ~15%, respectively), while in the latter ones, the second most abundant genus was *Serratia* (~15% relative abundance) at the end of shelf-life (185 h), even though the sporadic but high presence of *Shewanella* (~52% relative abundance) at the middle storage stage (88 h) should be also underlined.

Moreover, in order to highlight potential similarities and/or differences between the microbiota profile of different treatments, a PCoA was applied based on the weighted UniFrac distance (Figure S3). The analysis indicated a clear separation between the initial stage (0 h) and the advanced storage. Beyond that, at the end of shelf-life, both whole and filleted fish stored under aerobic conditions were grouped together, except for fillets stored at 12 °C, which were far from the other samples. In addition, the fillets stored under MAP conditions were another remarkable group, since according to PCoA plot, these samples were close to each other, separated from the other samples. However, it is crucial to mention that the fillets stored at 12 °C under MAP conditions were not included in this group, being separated from all samples. The main coordinates explained a total of 86.46% of the total variance (factors 1, 2 and 3 explained 56.21%, 19.33% and 10.92%, respectively).

## 4. Discussion

The determination of the microbiota profile, as well as the microbial changes of the European sea bass during storage at several different conditions, is the key to develop innovative and intelligent strategies to retard spoilage and thus, lead to the extension of product’s shelf-life. Nowadays, the fact that modern, reliable and high-throughput methods took the reins from the conventional culture-based microbiological techniques, has greatly contributed to the enrichment of our knowledge regarding the microbial communities’ changes of fish throughout its production line, from harvesting to storage-distribution until the end of shelf-life. Furthermore, the knowledge about the SSOs consortium that dominate at the end of shelf-life has been strengthened by the use of such an approach.

Sea bass’ shelf-life depends on a wide and complex range of factors, such as the initial microbial load, the initial composition of microbial communities, the microbial interactions, the storage conditions and so on. The combination of all the aforementioned parameters forms the profile that constitutes SSOs, which have the ability to deteriorate the sensorial attributes of fish via their metabolic activity, leading to product’s rejection from the consumers [7]. Combining and understanding all of these parameters would lead to develop a “biological network map” with invaluable impact for both scientific and industrial communities, in their attempt to tackle spoilage [6]. On that, the first and most important step is to obtain a holistic snapshot of fish microbial composition in different conditions. Thus, the study of sea bass’ microbiota profile and the changes that take place during commercial storage conditions, is always of great interest.

In the present study, according to the metataxonomic analysis, the initial microbial composition consisted of bacteria that are widespread in many ecosystems, including the aquatic one (*Pseudomonas*, *Thermus* and *Acinetobacter*, etc.). This finding is in line with the literature, since it has been noted that these bacteria are usually a major part of the initial microbiota of fresh fish [18,20,26,27], including the European sea bass [28,29,30]. Such bacteria mainly originate from fish habitats either seawater/freshwater or sediment [31], since they exhibit high resistance to a variety of environmental conditions, a fact that could explain their presence in fresh fish flesh. For instance, *Thermus* is a very common environment originating microorganism, whose resistance to extreme environmental temperatures and conditions is very well-known [32]. Furthermore, several species belonging to the genus *Acinetobacter* are usually found in fresh fish [33], since these bacteria are very abundant in aquatic ecosystem and easily colonize the skin and/or gills of fish [34]. Additionally, *Pseudomonas* has also been noted as a major part of fresh fish microbiota, especially on those from temperate waters [9,35,36] which is in perfect line with the present work.

As mentioned above, one of the main factors that affect the SSOs consortium that is responsible for product’s end of shelf-life is the initial microbiota. In our study, *Pseudomonas* was undoubtedly the dominant bacterium in the vast majority of treatments; especially on samples stored under aerobic conditions; not only in the fresh fish/fillets (0 h) but also throughout the storage period and at the end of each sample’s shelf-life. Indeed, *Pseudomonas* is one of the major SSOs found in air-stored spoiled fish from Hellenic waters [9,18,20,30,33,37,38,39]. This bacterium was also noted as a key spoiler player in several other fish species from different regions [40], as well as beyond Mediterranean area, in the Scandinavian region [41,42]. Apart from fish, *Pseudomonas* has also been detected at the spoilage point of several other aquatic products, including crab, shrimp and mussels [43,44,45], indicating that this bacterium plays a key role in spoilage of a plethora of seafood products.

Moreover, the noteworthy presence of *Acinetobacter* that followed the dominance of *Pseudomonas* in sea bass fillets stored at 12 °C, is also remarkable, suggesting that this bacterium is favored at higher storage temperatures. Indeed, according to Zotta et al., [40], *Acinetobacter* was found in high relative abundance in fish fillets stored at 10 °C. This bacterium has been previously detected in fish fillets, either at the middle [27] or at the end of shelf-life [46] as a part of bacterial consortium but not as the dominant microorganism, which is in perfect agreement with the present work. However, in a recent study by Du et al. [47], *Acinetobacter* was found to be the dominant player at the end of shelf-life of rainbow trout stored aerobically at 4 °C. At this point, it is crucial to mention that *Acinetobacter* is not recognized as a strong spoiler player at all [48,49]. Indeed, according to Liu et al. [50], this bacterium cannot hydrolyze fish proteins, being in parallel a weak producer of biogenic amines, as well as a weak degrader of ATP-related compounds.

Under a modified atmosphere (increased levels of CO_2_-decreased levels of O_2_), it has been previously reported that many bacterial groups (e.g., *Brochothrix*, *Serratia*, LAB, etc.) may prevail instead of the strictly aerobic and very CO_2_-sensitive *Pseudomonas* [36,41,51,52,53,54]. Furthermore, according to McMillin [55], Gram-negative bacteria are generally more sensitive to the presence of CO_2_ than Gram-positive ones, due to the fact that the majority of the latter bacteria are either obligate or facultative anaerobes. In the present work, although many of the bacteria belonging to such genera (e.g., *Carnobacterium*, *Brochothrix*) appeared under MAP conditions, their relative abundances did not reach the levels of *Pseudomonas* at storage temperatures of 0, 4 and 8 °C. This is in line with a previous study [28], where it has been reported that although under MAP conditions and low temperatures storage of whole or filleted sea bass the growth of *Brochothrix* and other LABs benefited, they were not able to surpass *Pseudomonas* levels. Thus, even though *Pseudomonas* has been proven to be a sensitive microorganism in the presence of CO_2_ by many studies [56,57,58], the findings of the present work may indicate a potential existence of facultative anaerobic species or even mutants *Pseudomonas* strains. The latter triggers the need for further study to this aspect (a) by isolating and in vitro studying different *Pseudomonas* strains from MAP-stored sea bass or (b) by an in-depth study at metagenomic level to reveal potential expressed genes and mechanisms that may be responsible for *Pseudomonas* resistance under such conditions.

Furthermore, it is also important to note the sporadic strong appearance of specific bacteria, at MAP-treated samples, at the middle phase of storage, followed by the noteworthy decrease of their abundance at the end of shelf-life. For instance, *Carnobacterium* appeared at high abundance in middle storage stage (377 h) of MAP-stored fillets at 0 °C. However, its abundance decreased significantly at the end of sample’s shelf-life. The same was observed in MAP-stored fillets at 8 °C, where at 88 h of storage *Shewanella* was the dominant microorganism, the relative abundance of which dramatically decreased at the end of the product’s shelf-life. Both bacteria have been previously reported as a vital part of spoiled seafood microbiota, e.g., for *Carnobacterium* in gilt-head sea bream stored at 8 °C [9] and for *Shewanella* in hake fillets [56]. Based on the above, it would be assumed that these bacteria play some kind of secondary role in the sea bass spoilage under specific MAP conditions, since their high presence in advanced storage point cannot be accidental. Those bacteria could be also considered as some kind of the so-called Ephemeral Spoilage Organisms (ESOs) [12,13], providing a specific contribution in spoilage course. The latter deserves more attention in the near future, since the concept of ESOs is not clearly defined. The lower relative abundances of such bacteria at the end of the respective product’s end of shelf-life could be attributed to a potential high competitiveness with other dominant players, in our case *Pseudomonas*, although the reasons for such a dramatic decrease are not clear and should be deeply investigated by further studies.

However, in higher than the aforementioned temperatures (12 °C) in MAP-stored samples, the microbiota profile was greatly influenced, indicating that a combination of modified atmosphere and high temperature contributes to a formation of different microbiota spoilage. In this case, *Serratia* was found to exhibit the highest relative abundance. As noted elsewhere, several species belonging to this genus can grow at a variety of storage conditions, including CO_2_-enriched anoxic atmospheres [59]. This genus has already been associated with the spoilage microbiota of seafood products, in particular in vacuum freshwater fish [60], in raw salmon [61,62,63] and in horse mackerel fillets stored under MAP [64] and has been correlated with high production of odors, as well as high amounts of trimethylamine (TMA) [53,63] and floor-cloth off-odors in cooked and peeled shrimps [65]. In a recent pan-genomic study by Begrem et al. [66], several genes of *Serratia* encoding mobile plasmids and transposases were detected. Authors suggested that those genes may help to acquire metabolic functions by horizontal gene transfer, useful for the survival of this microorganism during seafood storage. This is an understudied frontier, and undoubtedly, pan-genomics could be the key to elucidate the factors that favor *Serratia* dominance under specific conditions, as it happened in our work (MAP at 12 °C), as well.

Overall, a rational approach to the spoilage phenomenon requires a holistic study of microbial composition changes during sea bass storage. Many factors need to be considered in determining SSOs. Among others, handling, storage conditions and temperature are vital factors to achieve this aim. The present study demonstrates that all of these factors affect to some extent the onset time of SSOs’ growth (and consequently the end of the product’s shelf-life), while their final composition depends on storage conditions applied. Metabarcoding analysis is a rational and holistic approach to spoilage microbiota detection, revealing the presence and/or dominance of several bacteria that would be difficult to detect with conventional approaches. The present study complements and enriches the knowledge about the changes of whole and filleted sea bass microbial profile during different storge conditions, contributing to the attempt of developing specific strategies that could retard the growth of SSOs and thus, extend the shelf-life of sea bass. However, it would be an omission to forget that determining the microbiota profile is the first step towards obtaining the “big picture” on what is really happening during the spoilage course. Thus, further works, similar to the present study, are needed, but more importantly, other studies examining sea bass’ spoilage status at metagenomic and/or pan-genomic level are now needed as ever before, in order to proceed to the next level. Furthermore, as mentioned above, deep cooling is not ensured in real life neither in markets/restaurants nor in household refrigerators, mainly due to the constantly opening and closing that result in temperature rising to high levels. In this sense, the study of microbiota profile in a temperature range of 0–12 provides useful information, highlighting in parallel the need for storage conditions monitoring. Finally, the obtained data from the present work could be used (in combination with other similar works) in meta-analyses to develop reliable models to better predict the product’s shelf-life, depending on real storage conditions.

## 5. Conclusions

To ensure European sea bass quality, scientists attempt to describe its microbial diversity using modern molecular methods. Herein, 16S rRNA metabarcoding analysis highlighted several similarities and in specific cases some differences between different storage conditions, since in the majority of them, *Pseudomonas* was by far the dominant bacterium that may be responsible for deterioration of product’s sensorial attributes. However, it would be an omission not to highlight the dominance of other bacteria (*Serratia*) under specific conditions, as well as the sporadic presence of others at an advanced storage period. The role and contribution of such bacteria in the spoilage course should be deeply studied in the near future. The findings of the present work should be used as a benchmark in the attempt to provide to the international market seafood products with the longest shelf-life. To tackle such challenges, novel and intelligent strategies must be developed, and thus, further works are needed to enrich much more the existing knowledge about what really happens during fish spoilage in every stage of production (from aquaculture to harvesting and processing). This attempt would be further strengthened with the implementation of meta-genomics and pan-genomics. All the aforementioned will definitely lead to sea bass’ quality assurance “from farm to fork” in order to meet the currently increased consumer demand for high quality seafood.

## Figures and Tables

**Table 1:** Sea bass handling and storage conditions.

Sample ^2^	Atmosphere	Temperature (°C)
Whole Seabass	Air	0, 4, 8, 12
Filleted Seabass	Air	0, 4, 8, 12
Filleted Seabass	MAP ^1^	0, 4, 8, 12

^1^ MAP consisted of CO_2_, O_2_, N_2_, at levels of 31%, 23% and 46%, respectively, measured at the time of reception with Dansensor^®^ CheckPoint^®^ 3 (AMETEK MOCON, Minneapolis, MI, USA). ^2^ Number of subsamples stored under the different conditions (n = 30 from 3 lots for each treatment).

**Table 2:** Determination of products’ end of shelf-life, as assessed by sensory evaluation of five trained panelists.

Sample	Atmosphere	Temperature (°C)	End of Shelf-Life (h)
Whole Sea bass	Air	0	281
Whole Sea bass	Air	4	185
Whole Sea bass	Air	8	65
Whole Sea bass	Air	12	41
Filleted Sea bass	Air	0	143
Filleted Sea bass	Air	4	70
Filleted Sea bass	Air	8	58
Filleted Sea bass	Air	12	34
Filleted Sea bass	MAP	0	545
Filleted Sea bass	MAP	4	305
Filleted Sea bass	MAP	8	185
Filleted Sea bass	MAP	12	113

**Table 3:** Number of reads and alpha diversity indices of whole (SW) and filleted sea bass (SF) during storage (h) at various temperatures (C) under air (A) and MAP (M) conditions.

Sample	Raw Reads	Filtered Passing Reads	Observed Features ^1^	Shannon
SWA_0C_0h	4994	3685	76	4.89
SWA_0C_96h	20,085	13,930	135	5.82
SWA_0C_185h	20,709	9664	45	4.90
SWA_0C_281h	27,096	13,238	47	4.55
SWA_4C_41h	7255	4692	47	4.30
SWA_4C_135h	18,099	8600	35	4.49
SWA_4C_185h	15,564	6544	29	4.01
SWA_8C_41h	24,288	13,319	117	5.62
SWA_8C_65h	21,130	10,526	57	4.90
SWA_12C_23h	25,780	13,013	81	4.77
SWA_12C_41h	29,266	14,590	74	5.44
SFA_0C_0h	13,708	9355	114	5.75
SFA_0C_70h	58,857	32,042	50	4.00
SFA_0C_143h	23,662	15,583	93	4.87
SFA_4C_46h	30,998	15,236	67	4.96
SFA_4C_70h	33,789	17,742	65	4.63
SFA_8C_34h	16,466	7169	41	4.35
SFA_8C_58h	25,308	13,846	39	4.32
SFA_12C_28h	10,658	4227	25	4.04
SFA_12C_34h	22,962	12,020	49	4.97
SFM_0C_0h	7089	5845	56	4.16
SFM_0C_161h	19,552	9812	64	5.01
SFM_0C_377h	11,336	5188	24	3.57
SFM_0C_545h	23,868	12,248	30	3.66
SFM_4C_137h	36,548	15,649	50	4.78
SFM_4C_305h	37,086	19,233	53	4.56
SFM_8C_88h	37,098	15,620	53	4.84
SFM_8C_185h	37,291	20,632	46	4.55
SFM_12C_54h	34,161	14,363	52	5.13
SFM_12C_113h	46,307	17,838	50	4.87

^1^ Observed zero-radius Operational Taxonomic Units (zOTUs).

## Data Availability

The datasets generated for this study are available on request to the corresponding author.

## Supplementary Materials

The following supporting information can be downloaded at: https://www.mdpi.com/article/10.3390/microorganisms10091870/s1, Figure S1: Shannon–Wiener rarefaction curves of whole (SW) and filleted sea bass (SF) at intervals of storage hours (h) at various temperatures (C) under air (A) and MAP (M) conditions, Figure S2: Relative abundance (%) of bacterial phyla of whole (SW) and filleted sea bass (SF) at intervals of storage hours (h), at various temperatures (C) under air (A) and MAP (M) conditions, as revealed by 16S rRNA metabarcoding analysis; Figure S3: Principal Coordinate Analysis (PCoA) plot based on weighted UniFrac distance. Different color corresponds to whole (SW) and filleted sea bass (SF) at intervals of storage hours (h), at various temperatures (C) under air (A) and MAP (M) conditions.
