# Supplementary material for: Microbiota Succession of Whole and Filleted European Sea Bass (Dicentrarchus labrax) during Storage under Aerobic and MAP Conditions via 16S rRNA Gene High-Throughput Sequencing Approach
Source: Microorganisms. 2022 Sep 19;10(9):1870. doi: 10.3390/microorganisms10091870 (PMC9505548; doi:10.3390/microorganisms10091870)
Supplement: Supplementary file 1 [file microorganisms-10-01870-s001.zip › microorganisms-1876958-supplementary.pdf]

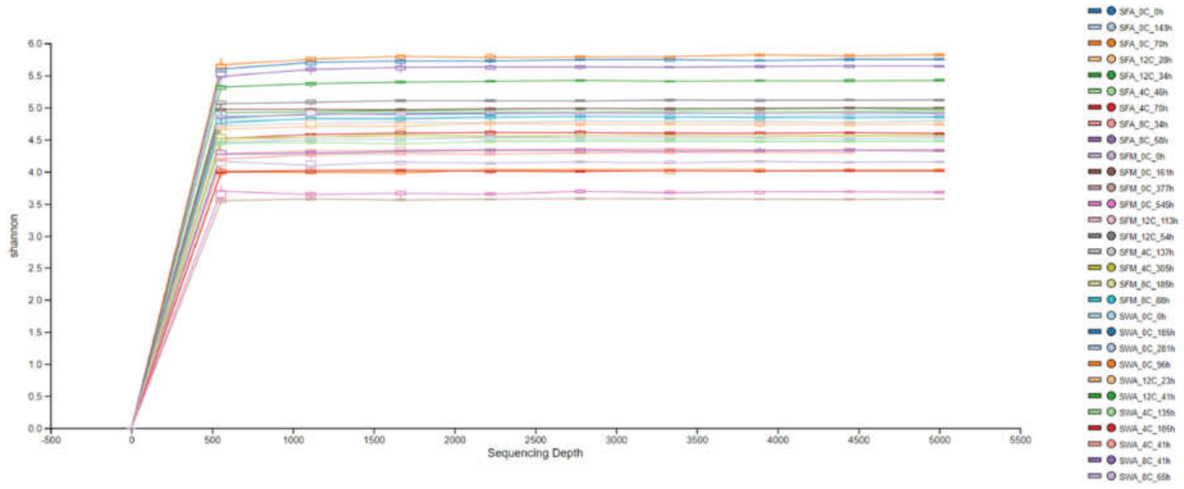

Figure S1. Shannon-Wiener rarefaction curves of whole (SW) and filleted sea bass (SF) at intervals of storage hours (h) at various temperatures (C) under air (A) and MAP (M) conditions.

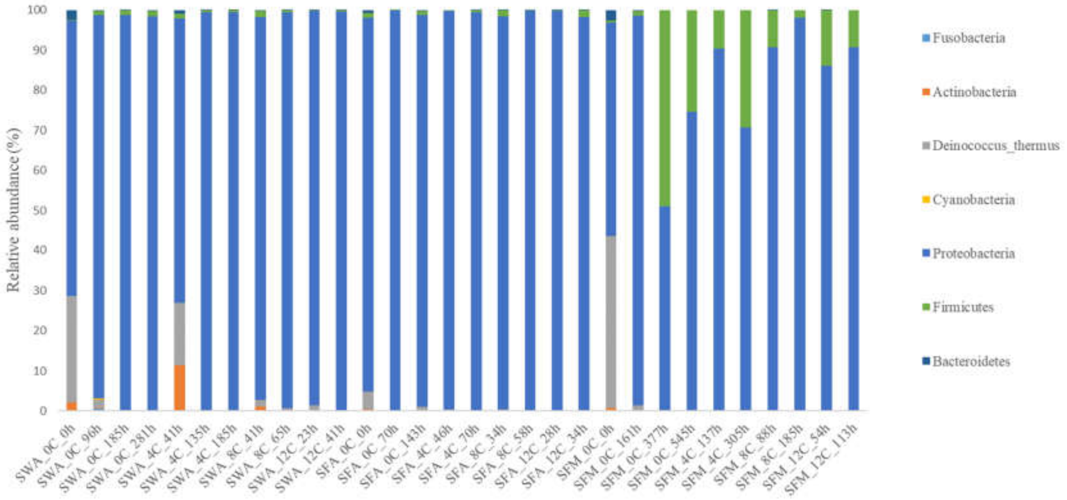

Figure S2. Relative abundance (%) of bacterial phyla of whole (SW) and filleted sea bass (SF) at intervals of storage hours (h), at various temperatures (C) under air (A) and MAP (M) conditions, as revealed by 16S rRNA metabarcoding analysis.

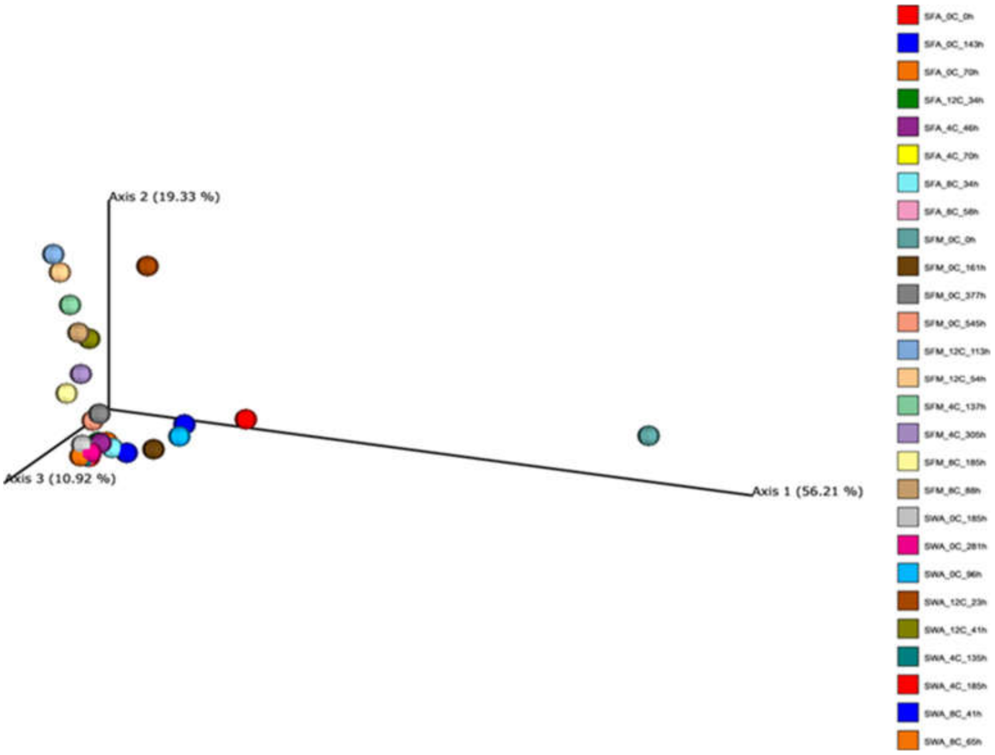

Figure S3. Principal Coordinate Analysis (PCoA) plot based on weighted UniFrac distance. Different color corresponds to whole (SW) and filleted sea bass (SF) at intervals of storage hours (h), at various temperatures (C) under air (A) and MAP (M) conditions.
